# Thyroid cancer associated with Hashimoto thyroiditis: similarities and differences in an endemic area

**DOI:** 10.1186/s43046-020-0017-9

**Published:** 2020-01-17

**Authors:** Osama Hussein, Khaled Abdelwahab, Omar Hamdy, Shadi Awny, Nermin A. Megahed, Mohamed T. Hafez, Amr F. Elalfi, Mahmoud Abdelaziz, Khaled Gaballah, Mohamed Abdelkhalek

**Affiliations:** 1 Surgical oncology unit, Mansoura University Oncology center, Mansoura, Egypt; 2 Pathology department, Mansoura University Faculty of Medicine, Mansoura, Egypt

**Keywords:** Thyroid cancer, Hashimoto’s thyroiditis, Papillary thyroid carcinoma, Endemic goiter, Egypt

## Abstract

**Background:**

Hashimoto thyroiditis (HT) is an autoimmune lymphocytic thyroiditis and is the most common form of thyroid inflammatory diseases. The association of HT with papillary thyroid carcinoma (PTC) has been described. PTC is the most common form of malignancy associated with HT. When papillary carcinoma develops on top of Hashimoto thyroiditis, the disease tends to be less aggressive and lymph node and extra-thyroidal invasion are infrequent.

**Results:**

We retrospectively examined the pathological features of our patients who were diagnosed with concomitant HT and thyroid cancer. In Egyptian patients, PTC was the main type of malignancy associated with HT (96.2%) and was often multifocal (46.2%). In contrast to the published literature, lymph node invasion and extra-thyroidal extension were as frequent in association with HT as in other cancer cohorts. We also observed the frequent occurrence of Hürthle cell metaplasia (23.1%) and the appreciable incidence of aggressive histological types of PTC (32%).

**Conclusion:**

Thyroid carcinoma with HT may have some aggressive features in areas with endemic goiter background.

## Background

Papillary thyroid carcinoma (PTC) is the most common form of differentiated thyroid cancer (DTC) [1, 2]. Several risk factors are associated with increased incidence of PTC including iodine sufficiency and autoimmune lymphocytic thyroiditis—widely known as Hashimoto’s thyroiditis (HT). Hashimoto’s disease is the most common type of thyroiditis and similar to PTC; it is more prevalent in iodine-replete regions [3–5]. The relation of Hashimoto’s disease to papillary carcinoma has been comprehensively described in the literature [6–16]. In patients with HT, papillary carcinoma is more frequent and less aggressive. The association between the two disease entities is well established. However, the existence of a causal relation has always been debated. Both diseases share several epidemiologic features including relative high prevalence, female predominance and predilection to iodine replete locations. Amplification of rearranged during transfection (RET) oncogene has been described in Hashimoto’s patients [17, 18]. It remains however unclear whether HT plays a definitive causative role in the PTC carcinogenesis. PTC that arises on top of HT shows several indicators of a relatively good prognosis [8, 9, 12, 16, 19]. Hashimoto-associated papillary carcinoma frequently develops in younger females and has smaller nodule size and less incidence of lymph node invasion. In spite of the elaborate description of the Hashimoto-associated papillary cancer in the literature, our knowledge of this disease variant originates largely from non-endemic populations. Endemic areas of iodine deficiency have their characteristic epidemiologic and histological patterns of thyroid cancer. How iodine deficiency modulates the relation of papillary carcinoma to Hashimoto’s thyroiditis has not been investigated before. In this report, we describe the pathological features of Egyptian patients diagnosed with PTC on top of HT. We compare these to a published cohort of thyroid cancer from the same institution (Ahmed and Abolenaga 2015) [20] in order to identify the characteristics of the papillary-Hashimoto association in an endemic population.

## Methods

We retrospectively reviewed the files of all Hashimoto’s disease specimens performed at our Center from June 2012 to June 2016. All patients’ files with histological diagnosis of lymphocytic thyroiditis in the postoperative pathology were examined for the presence of carcinoma. Hashimoto-associated thyroid cancer in this cohort was compared to a published dataset of thyroid cancer from our institution. The published dataset included all thyroid cancers at our University Hospitals from January 2003 to December 2011. Disease characteristics were compared using Fisher exact and Mann-Whitney tests.

## Results

Twenty-four percent of patients admitted to the oncology center for thyroidectomy had Hashimoto’s disease. Of all patients with lymphocytic thyroiditis, 26 patients had associated thyroid carcinoma. We compared these patients to the published dataset of thyroid cancer from our institution (Ahmed and Abolenaga 2015) [20].

In comparison to thyroid cancer in general, patients with Hashimoto-associated cancer were more frequently females (68.8% vs. 96.2% respectively; *p* = 0.0016). Although Hashimoto-associated patients tended to be younger, there was no significant age difference between the two groups.

**Fig. 1:**
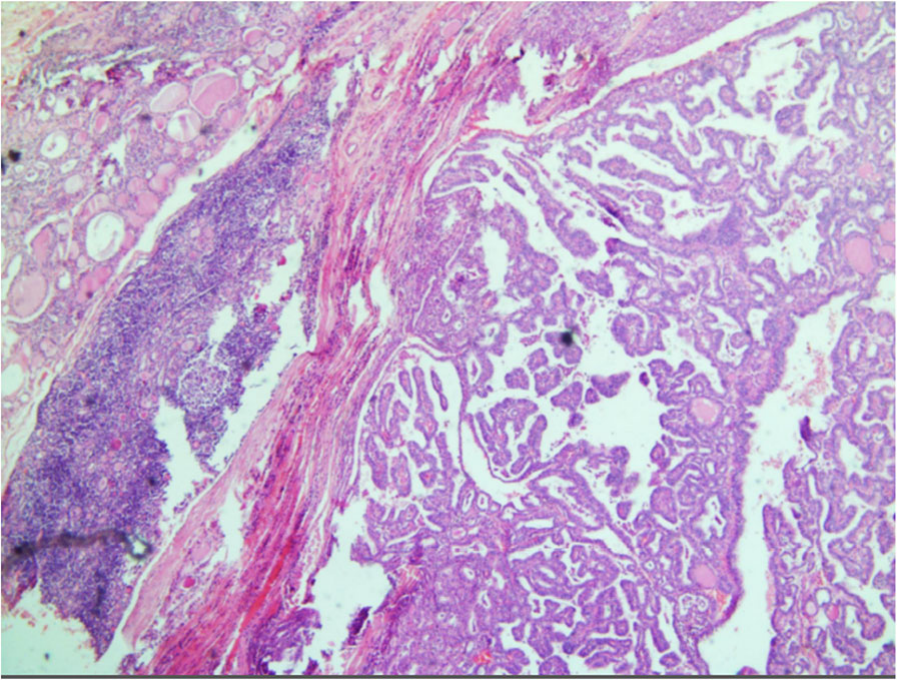
Papillary thyroid carcinoma (right) on background of lymphocytic thyroiditis (left) (H&E × 40)

Papillary carcinoma was the most common form of thyroid cancer in association with lymphocytic thyroiditis (Fig. 1). Papillary histology formed 96.2% of thyroiditis-associated cancer specimen while this form of cancer accounted for only 74.1% of thyroid cancer in our locality (*p* = 0.0093).

Multifocality was observed in 46.2% of thyroiditis-associated cancer. The high incidence of multifocality contrasted with 6.5% generally observed in thyroid carcinoma at our university practice (*p* = 0.0001).

There was no significant difference in the rate of cervical lymph node involvement in thyroiditis-associated carcinoma compared to thyroid cancer in general (38.5% and 37.5% respectively; *p* = 1.0000). Similarly, there was no significant difference in the rate of extra-thyroidal extension (15.4% and 7.8%, respectively; *p* = 0.1493) (Table 1).

**Table 1:** Thyroid cancer with Hashimoto thyroiditis compared to thyroid cancer without HT

	Cancer without HT Published cohort (ref. 19) (total no. = 644) *N* (%)	Cancer with HT Present study cohort (total no. = 26) *N* (%)	*P* value
Female gender	443 (68.8)	25 (96.2)	0.0016
Papillary pathology	477 (74.1)	25 (96.2)	0.0093
Positive LNs	243 (37.5)	10 (38.5)	1.0000
Extra-thyroidal extension	50 (7.8)	4 (15.4)	0.1493
Multifocality	42 (6.5)	12 (46.2)	0.0001

Of these Hashimoto-associated PTC, we observed certain histolological features indicative of unfavorable disease outcome. Background Hürthle cell metaplasia was observed in 23% of the Hashimoto-associated cancer. Moreover, this group included several cases of aggressive papillary carcinoma variants: two oncocytic (Hürthle) cell variant, four follicular variant, one de-differentiated variant, and one tall columnar cell variant (Table 2).

**Table 2:** Additional observations in cancer with Hashimoto thyroiditis

Papillary carcinoma with Hashimoto thyroiditis		*N* (%)
Background Hürthle cell metaplasia		6 (23.1)
Special variants of papillary carcinoma N (% of all PTC)	De-differentiated	1 (4)
	Columnar	1 (4)
	Follicular	4 (16)
	Oncocytic (Hürthle)	2 (8)

## Discussion

The Nile valley of Egypt is characteristically an endemic area of iodine deficiency goiter [21, 22]. The endemic nature of the region casts certain features on the thyroid cancer profile in our locality. At our University, follicular carcinoma constituted 12.6% of thyroid cancer patients [20]. Papillary carcinoma remained the most common variant with a modest frequency of 74.1%. It is widely believed that iodine repletion increases the incidence of both papillary carcinoma of the thyroid as well as Hashimoto`s thyroiditis. In iodine-replete regions, both diseases are prevalent [3-5]. The association between the two thyroid disease entities has been elaborately discussed based on retrospective analysis of patient populations in these iodine-sufficient regions. In our cohort, thyroiditis-associated cancer showed many of the features described in the literature. In others, Egyptian patients significantly deviated from that description.

Hashimoto-associated thyroid cancer is almost always of the papillary type. Papillary carcinoma on top of Hashimoto`s thyroiditis is known to be a less aggressive disease. Several markers of good prognosis of PTC are specifically associated with the presence of Hashimoto`s thyroiditis. In patients with HT, papillary carcinoma tends to be a disease of younger age and the primary tumor is often small. Although multifocality of PTC is characteristic in thyroid with Hashimoto`s thyroiditis, lymph node involvement and extra-thyroidal extension are infrequently observed. These features have been consistently described in several large retrospective studies and meta-analyses [3, 7–10, 12–16, 19]. Lee et al. analyzed the data of 38 published studies addressing PTC with HT [9]. The meta-analysis included a total of 10,648 patients. Major findings of this meta-analysis reciprocated the generally observed trend in the English literature and were used to compare our Egyptian patients with the global pattern.

In our and others’ experience, PTC is the predominant form of cancer in Hashimoto`s thyroids. In our practice, papillary carcinoma constituted 96.2% of cancer in HT patients while this form of cancer accounted for 74.1% of all thyroid cancer cases. In the meta-analysis published by Lee and colleague, HT was associated with PTC more than any other form of thyroid cancer (OR = 2.432; 95% CI = 1.614–3.665; *p* = 0.001) [9]. Similarly, our data corroborated the findings of Lee and colleagues regarding female preponderance, multifocality, and lack of association with young age. In their data, being a female and having a multifocal tumor was significantly associated with the presence of HT (OR = 2.678; 95% CI = 1.755–4.087; *p* = 0.001 and OR = 1.467; 95% CI = 1.096–1.964; *p* = 0.010; respectively) [9].

In contrast to the published literature, we observed some characteristic features of aggressive behavior of thyroid carcinoma associated with Hashimoto’s disease. Our patients had similar risk of lymph node involvement and extra-thyroidal extension whether they had associated HT or not. This is in contrast to the generally held idea of HT-associated PTC being a disease of low risk of nodal and extra-thyroidal involvement. Similar to several other authors, Lee and colleagues reported that PTC with HT was related to the absence of lymph node metastases and the lack of extra-thyroidal extension (OR = 1.287; 95% CI = 1.010–1.639; *p* = 0.041 *and* OR = 1.295; 95% CI = 1.098–1.527; *p* = 0.002; respectively) [9].

Some histological clues also pointed that HT association with PTC may portend aggressive behavior in Egyptian patients. We observed a high prevalence of Hürthle cell metaplasia and several incidences of aggressive histological variants of PTC associated with lymphocytic thyroiditis. Oncocytic cell, tall columnar cell, and follicular and de-differentiated variants are established markers of aggressive papillary carcinoma behavior that were observed in our cohort of Hashimoto-associated papillary carcinoma.

Although few recent reports pointed to some aggressive features of thyroid carcinoma in pediatric population affected with lymphocytic thyroiditis [23, 24], the generally held concept is that HT predicts a better prognosis of associated thyroid cancer.

In the current study, we clearly identified some peculiar pathologic features of thyroid carcinoma associated with lymphocytic thyroiditis. We suggest these peculiarities may at least in part be attributed to the endemic nature of the locality and the background iodine deficiency. In view of this small retrospective sample and in the absence of survival or mechanistic information, more evidence is required to complete the missing parts of the picture.

## Conclusions

In our experience, patients with lymphocytic thyroiditis and cancer had several pathological features of aggressive disease that have not been hitherto described. These observations suggest wider interaction between iodine supply, autoimmunity, and carcinogenesis.

## Data Availability

All the clinical, radiological, and pathological data used in this manuscript is available on Mansoura University medical system (Ibn Sina Hospital management system). http://srv137.mans.edu.eg/mus/newSystem/

## Abbreviations

HT: Hashimoto thyroiditis
PTC: Papillary thyroid carcinoma
RET: Rearranged during transfection
